# Short Communication: Efficacy of Two Commercial Disinfectants on *Paenibacillus larvae* Spores

**DOI:** 10.3389/fvets.2022.884425

**Published:** 2022-05-12

**Authors:** Joseph Kiriamburi, Jamleck Muturi, Julius Mugweru, Eva Forsgren, Anna Nilsson

**Affiliations:** ^1^Department of Biological Sciences, University of Embu, Embu, Kenya; ^2^Department of Ecology, Swedish University of Agricultural Sciences, Uppsala, Sweden

**Keywords:** *Paenibacillus larvae*, American foulbrood, disinfectants, apis mellifera, spores

## Abstract

*Paenibacillus larvae* is a spore-forming bacterium causing American foulbrood (AFB) in honey bee larvae. The remains of a diseased larva contains billions of extremely resilient *P. larvae* spores viable for decades. Burning clinically symptomatic colonies is widely considered the only workable strategy to prevent further spread of the disease, and the management practices used for decontamination requires high concentrations of chemicals or special equipment. The aim of this study was to test and compare the biocidal effect of two commercially available disinfectants, “Disinfection for beekeeping” and Virkon S on *P. larvae*. The two products were applied to *P. larvae* spores in suspension as well as inoculated on two common beehive materials, wood and Styrofoam. “Disinfection for beekeeping” had a 100 % biocidal effect on *P. larvae* spores in suspension compared to 87.0–88.6% for Virkon S which, however, had a significantly better effect on *P. larvae* on Styrofoam. The two disinfectants had similar effect on infected wood material.

## Introduction

*Paenibacillus larvae* is a spore-forming, Gram-positive bacterium causing the severe disease American foulbrood (AFB) in honey bee larvae. Honey bee larvae become infected from ingesting food contaminated with *P. larvae* spores that germinate in the midgut and eventually kills the larvae. The remains of the larvae contains billions of spores and serves as sources for new infections. The *P. larvae* spores are resilient and can remain viable in the environment for decades (1–3). A common way to control AFB is by burning the contaminated hives and bees, although the latter can sometimes be saved as an artificial swarm, housed on new or disinfected material (4). Hive material can be decontaminated using chemical disinfectants or heat. Chemical disinfectants have been shown to have a high efficacy on spores in suspension, but less effective on wood-based equipment (3, 5). There are several methods using heat for decontamination of hive material, for example dipping in hot paraffin, scorching, dry heat and autoclaving (3). These methods are effective (3, 6), but requires access to advanced equipment.

Our aim was to test and compare the biocidal effect of 2 disinfectants, “Disinfection for beekeeping” (DFB) (Swienty, Denmark) and Virkon S (Lanxess, Germany) on *P. larvae* spores. DFB is developed for disinfection of hive material, gloves and tools and, according to the manufacturer (www.swienty.com, viewed October 9 2019), have a 99.99% biocidal effect on all viruses, bacteria, spores and fungi. Virkon S is a common disinfectant that have been on the market for over 30 years, originally developed for farm and livestock production (7) (www.virkons.se, viewed October 8 2019).

## Materials and Methods

A spore suspension was prepared from *P. larvae* cultures on agarplates (14 days to obtain sporulation) in sterile 0.9% saline solution. The spore suspension was stored at 4°C, heat shocked at 85°C for 10 min and diluted to the desired concentrations before the start of each experiment.

**Figure 1:**
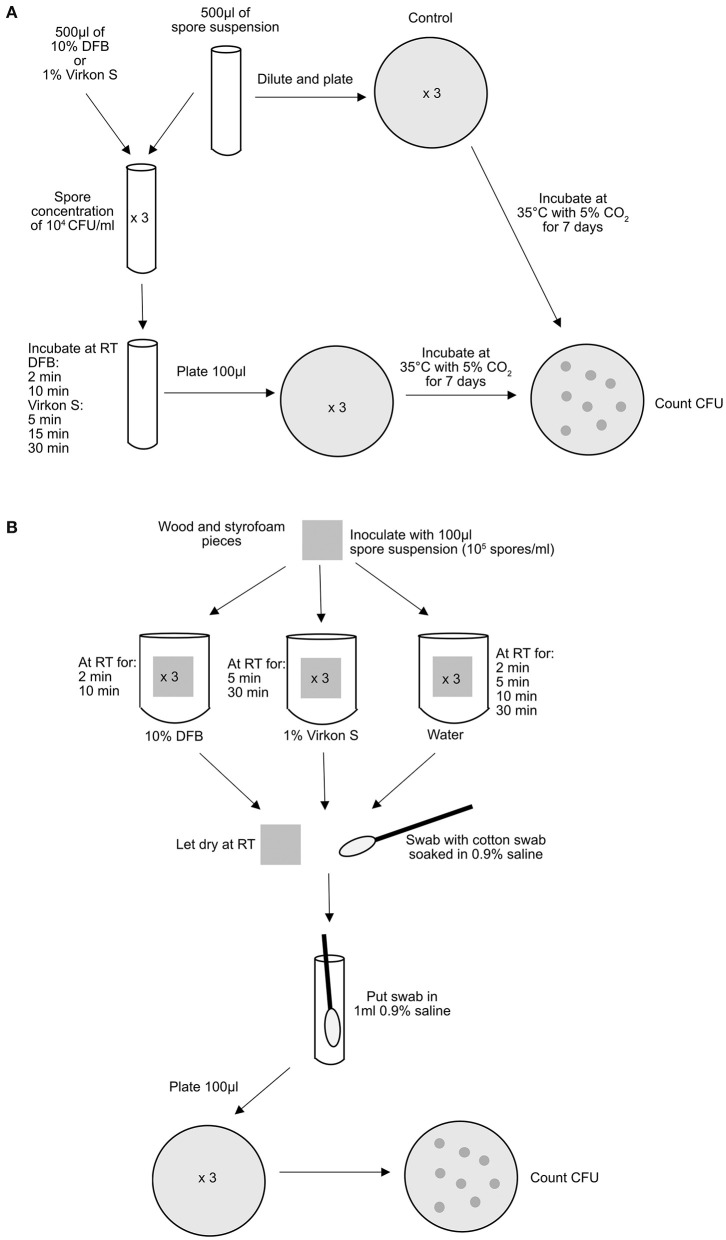
Schematic description of the experimental setup. **(A)** in spore suspension and **(B)** on wood and Styrofoam. RT, room temperature.

The experiments were performed as described in Figure 1 and repeated at least 3 times. *P. larvae* were cultured according to standard cultivation methods (8).

The biocidal effect of the disinfectants was calculated by comparing the number of CFUs from the treated samples and the untreated spore suspension or the mock treated wood and Styrofoam pieces.

Student's *t* test (unpaired, 2-tailed) was used to identify statistically significant differences, with a *P* ≤ 0.05 considered significant.

## Results

**Figure 2:**
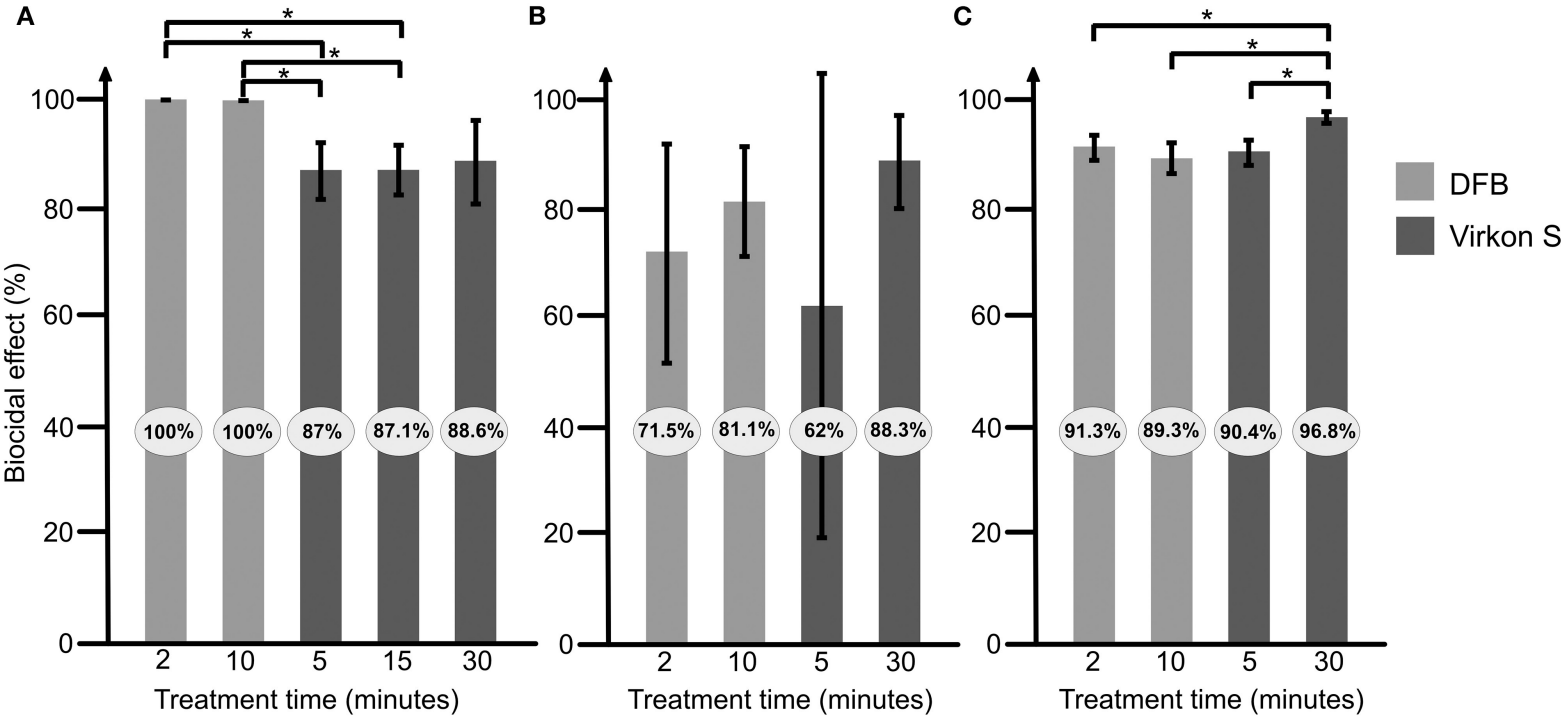
Efficacy of the 2 disinfectants “Disinfection for beekeeping” (DFB) and Virkon S on *Paenibacillus larvae* spores in suspension **(A)**, on wood **(B)**, and on Styrofoam **(C)**. Light gray bars shows results for DFB and darker gray Virkon S. The result is presented as an average from at least 3 repeats, with error bars indicating standard deviation. Significant differences are indicated. *P <0.05.

DFB had the highest biocidal effect (100% already after 2 min) on spores in suspension and was significantly more efficient than the 5 and 15 min Virkon S treatments (all *P* = 0.01, Figure 2A).

On wood, no significant differences could be seen between DFB and Virkon S, or the between the different treatment times (Figure 2B).

On Styrofoam, a significantly higher biocidal effect was observed after 30 min treatment with Virkon S compared to 2 and 10 min treatment with DFB (both *P* = 0.02, Figure 2C). The 30 min treatment with Virkon S had also a significantly higher biocidal effect than the 5 min treatment (*P* = 0.01, Figure 2C).

## Discussion

This study compares the biocidal effect of 2 disinfectants on *P. larvae* spores. Both disinfectants had an effect on the bacterial spores in suspension and on wood and Styrofoam. DFB had the best effect on the bacterial spores in suspension where all *P. larvae* spores were killed. These results are in line with the information from the manufacturer saying that DFB kills all viruses, bacteria, fungi and spores within 45 s. However, the effect of DFB on spores on wood and Styrofoam was lower than in suspension (Figure 1). Virkon S was slightly less effective than DFB on spores in suspension, but the differences were not significant. Thirty minutes treatment (recommended by the manufacturer) of Virkon S on contaminated Styrofoam was significantly more effective than the treatment with DFB (Figure 2C). Virkon S has in a previous study been shown to kill 80% of *P. larvae* spores (9). In this study however, the biocidal effect ranged from 88.6 to 96.8% after 30 min treatment (Figure 1). The effect of both disinfectants on wood varied more than the effect on Styrofoam and in suspension, most likely due to difficulties recovering *P. larvae* from wood. This is probably because wood is more porous and absorbs the liquid with the spores. *P. larvae* spores can “hide” in wood, making it more difficult for the disinfectant to access the bacterium. The wood and Styrofoam pieces used in this study were clean, i.e., they were not covered in wax or propolis. Any disinfectants will probably be less effective on used, non-cleaned hive material where large amounts of bacterial spores may be inaccessible to the disinfectants. It is therefore important that infected materials are thoroughly cleaned before being treated with disinfectants.

## Data Availability Statement

The original contributions presented in the study are included in the article/supplementary material, further inquiries can be directed to the corresponding author.

## Author Contributions

EF and AN developed the research concept. JK, EF, and AN designed and performed the experiments. JK, JMut, JMug, and AN co-wrote the manuscript. EF provided the resources, supervision, and funding assistance. All authors contributed to the article and approved the submitted version.

## Conflict of Interest

The authors declare that the research was conducted in the absence of any commercial or financial relationships that could be construed as a potential conflict of interest.

## Publisher's Note

All claims expressed in this article are solely those of the authors and do not necessarily represent those of their affiliated organizations, or those of the publisher, the editors and the reviewers. Any product that may be evaluated in this article, or claim that may be made by its manufacturer, is not guaranteed or endorsed by the publisher.

## References

[1] Forsgren E Stevanovic J Fries I. Variability in germination and in temperature and storage resistance among *Paenibacillus larvae* genotypes. Vet rinary Microbiol. (2008) 129:342–9. 10.1016/j.vetmic.2007.12.001 18207338

[2] Haseman L. How long can spores of american foulbrood live? Am Bee J. (1961) 101:298–9.

[3] Dobbelaere W. Disinfection of wooden structures contaminated with *Paenibacillus larvae* subsp. Larvae spores. J Appl Microbiol. (2001) 91:212–216. 10.1046/j.1365-2672.2001.01376.x 11473585

[4] Genersch E. American Foulbrood in honeybees and its causative agent, *Paenibacillus larvae. J Invertebrate Pathol*. (2010) 103:S10–9. 10.1016/j.jip.2009.06.015 19909971

[5] Okayama A Sákogawa T Nakajima C Hayama T. Sporicidal activities of disinfectants on *Paenibacillus larvae. J Vet Med Sci*. (1997) 59:953–4. 10.1292/jvms.59.953 9362051

[6] Del Hoyo M Basualdo M Torres J Bedascarrasbure E. Use of DHT-Equpment for Disinfection of AFB-Contaminated Beehive Materials in Argentina. Am Bee J. (1998) 138:738–40.

[7] Hernández A. Assessment of in-vitro efficacy of 1% Virkon® against bacteria, fungi, viruses and spores by means of AFNOR guidelines. Journal of Hospital Infection. (2000) 46:203–209. 10.1053/jhin.2000.0818 11073729

[8] Nordström S Fries I. A comparison of media and cultural conditions for identification of Bacillus larvae in honey. J Apic Res. (1995) 34:97–103. 10.1080/00218839.1995.11100894

[9] Hansen H Brødsgaard CJ. American foulbrood: a review of its biology, diagnosis and control. Bee World. (1999) 80:5–23. 10.1080/0005772X.1999.1109941524761740

